# Early Dendritic Morphogenesis of Adult-Born Dentate Granule Cells Is Regulated by FHL2

**DOI:** 10.3389/fnins.2020.00202

**Published:** 2020-03-17

**Authors:** Afrinash Ahamad, Jia Wang, Shaoyu Ge, Gregory W. Kirschen

**Affiliations:** ^1^Graduate Program in Neuroscience, Stony Brook University, Stony Brook, NY, United States; ^2^School of Health Technology and Management, Stony Brook University, Stony Brook, NY, United States; ^3^Biomedical Pioneering Innovation Center, Peking University, Beijing, China; ^4^Department of Neurobiology and Behavior, Stony Brook University, Stony Brook, NY, United States; ^5^Medical Scientist Training Program (MSTP), Renaissance School of Medicine at Stony Brook University, Stony Brook, NY, United States

**Keywords:** dentate gyrus, hippocampal neurogenesis, four and a half LIM domain protein 2, sphingosine 1 phosphate (S1P), dendritic growth

## Abstract

Dentate granule cells (DGCs), the progeny of neural stem cells (NSCs) in the sub-granular zone of the dentate gyrus (DG), must develop and functionally integrate with the mature cohort of neurons in order to maintain critical hippocampal functions throughout adulthood. Dysregulation in the continuum of DGC development can result in aberrant morphology and disrupted functional maturation, impairing neuroplasticity of the network. Yet, the molecular underpinnings of the signaling involved in adult-born DGC maturation including dendritic growth, which correlates with functional integration, remains incompletely understood. Given the high metabolic activity in the dentate gyrus (DG) required to achieve continuous neurogenesis, we investigated the potential regulatory role of a cellular metabolism-linked gene recently implicated in NSC cycling and neuroblast migration, called Four and a half LIM domain 2 (FHL2). The FHL2 protein modulates numerous pathways related to proliferation, migration, survival and cytoskeletal rearrangement in peripheral tissues, interacting with the machinery of the sphingosine-1-phosphate pathway, also known to be highly active especially in the hippocampus. Yet, the potential relevance of FHL2 to adult-born DGC development remains unknown. To elucidate the role of FHL2 in DGC development in the adult brain, we first confirmed the endogenous expression of FHL2 in NSCs and new granule cells within the DG, then engineered viral vectors for genetic manipulation experiments, investigating morphological changes in early stages of DGC development. Overexpression of FHL2 during early DGC development resulted in marked sprouting and branching of dendrites, while silencing of FHL2 increased dendritic length. Together, these findings suggest a novel role of FHL2 in adult-born DGC morphological maturation, which may open up a new line of investigation regarding the relevance of this gene in physiology and pathologies of the hippocampus such as mesial temporal lobe epilepsy (MTLE).

## Introduction

The discovery of mammalian adult hippocampal neurogenesis has been fundamental to our understanding of physiological homeostasis in the adult brain (Altman and Das, 1965). Most recently, studies of cellular metabolism and pharmacology have shown that lipid metabolism and signaling play an active role in adult neural stem cell cycling and are important for synaptic homeostasis and functional regulation of newborn neurons (Kanno and Nishizaki, 2011; Riganti et al., 2016; Beckervordersandforth et al., 2017; Stessin et al., 2017; Callihan et al., 2018). In particular, sphingosine-1-phosphate (S1P), well-known for its effects on cellular survival, proliferation, and motility in cancer biology and immunology, has recently been found to signal in the hippocampal dentate gyrus via various S1P receptors (S1PRs), controlling levels of neurogenesis (Matloubian et al., 2004; Milstien and Spiegel, 2006; Pyne and Pyne, 2010; Efstathopoulos et al., 2015; De Marco et al., 2017). However, the downstream effectors and intracellular machinery involved in the regulation of adult hippocampal neurogenesis remain largely unexplored.

Interestingly, a previous unbiased transcriptional analysis of human patients with mesial temporal lobe epilepsy (MTLE), a condition characterized by aberrant hippocampal discharge related to dysregulated neurogenesis, revealed a candidate gene linked to the S1P pathway that was markedly downregulated in hippocampi of patients with MTLE versus those without MTLE (Jamali et al., 2006; Parent and Murphy, 2008; Sierra et al., 2015). The gene in question is Four and a Half LIM domain protein 2 (FHL2), which codes for an adapter protein that mediates a variety of protein-protein interactions. FHL2 modulates numerous signaling pathways including cell proliferation and migration, with its conserved zinc finger motif allowing it to serve as a central hub for diverse signaling pathways to converge (Wixler et al., 2000). For instance, in cell culture S1P triggers RhoA GTPase activation followed by nuclear translocation of FHL2, where it acts as a transcriptional co-regulator for a number of genes including those involved in molecular memory and extracellular communication (Muller et al., 2002). Despite the finding of changes in FHL2 expression in subjects with MTLE (Jamali et al., 2006), surprisingly little follow-up on mechanistic characterization has taken place, though the authors speculated that the physical association of FHL2 to a potassium channel subunit may predispose these patients to epilepsy via a channelopathy. One recent study identified an alternative action of FHL2 expressed in adult neural stem cells (NSCs) that may help explain its clinical phenotype and physiological importance in the brain. Kim et al. reported that global FHL2 deletion in mice via transgenic constitutive knockout led to low self-renewal activity among NSCs, premature differentiation into astrocytes at the expense of neuronal differentiation and delayed neuroblast migration in the developing brain (Kim et al., 2019).

To determine the specific role of FHL2 in adult hippocampal neurogenesis while avoiding developmental effects from constitutive expression and non-specific effects from communicating adjacent cells, we thus aimed to carry out cell-autonomous and temporally controlled manipulation of FHL2 expression in adult-born dentate granule cells (DGCs). Through virally mediated genetic silencing and overexpression studies, we found that FHL2 is critical for dendrite growth in the early phase of newborn neuron development in the adult dentate gyrus.

## Materials and Methods

All experimental and surgical procedures were approved by the Stony Brook University Animal Use Committee and followed the guidelines of the National Institutes of Health (NIH).

### Animals

Eight-week-old male and female C57BL/6 wild type mice (Charles River Laboratories) were used in the protocol as approved by the Institutional Care and Animal Use Committee of Stony Brook University. All mice were housed in a cage on a 12-h light/dark cycle and were provided *ad libitum* access to food and water.

### Viruses

Retroviral and lentiviral production was performed as we previously described (Gu et al., 2012; Kirschen et al., 2017). The inducible FHL2 shRNA vector was constructed as follows: pSiREN-Tet-shRNA-FHL2-Flex-i(tTS-P2A-eGFP), and the GFP-only vector was constructed as follows: pSiREN-Tet-shRNA-Luc-Flex-i(tTs-P2A-eGFP). The FHL2 overexpression vector was constructed as follows: pUX-FHL2-mRFP.

### Doxycycline Induction

Transgenes were activated with the administration of doxycycline in drinking water. The drinking water included 400 mg doxycycline dissolved in 200 mL distilled water plus 30 g of sucrose.

### 5- Bromo-2′ Deoxyuridine (BrdU) Administration

A single dosage of 10 μl/g of BrdU was administered intraperitoneally (i.p.) and mice were euthanized 1 day, 5- and 14-days post-injection. Each time point included 3 animals per group.

### Surgeries

All surgeries were performed aseptically. Before surgery mice were first weighed and anesthetized with 200 mg/kg of ketamine/xylazine administered intraperitoneally. Retrovirus was infused, or lentivirus and retrovirus (lenti-GFAP-Cre (retro-Flex-reverse-eGFP or retro-Flex-reverse-shFHL2) were co-infused into the DG (0.5 μL/injection site) at stereotactic coordinates -2.0 mm from bregma, ±1.6 mm lateral, -0.25 mm ventral, and -3.0 mm from bregma, ±2.6 mm lateral, -0.32 mm ventral. Mice were monitored during the intraoperative and postoperative duration and a heating pad (37 C) was provided for 2 h during the recovery phase. Also, mice were given 0.05 mg/kg of buprenorphine HCl intraperitoneally as post-operative recovery analgesic.

### Perfusion and Tissue Processing

Mice were anesthetized with urethane (200 μg/g) and were perfused transcardially with Phosphate Buffered Saline (PBS) and then 4% Paraformaldehyde (PFA). Brains were removed and fixed in 4% PFA for 24 h at 4^0^C and were transferred following day to 30% (w/v) sucrose solution. The brains were sectioned coronally at 60 μm on Leica microtome. Three-fourths of the sections were stored in cryopreservative (30% sucrose, 30% glycerol by weight in deionized water) until future staining and a quarter of the sections were immediately washed 3x with PBS before immunostaining.

### Immunohistochemistry

For BrdU labeling experiments, sections were pretreated with 2N HCL at 37^0^ C for 15 min followed by 10 min in 0.1 M Borate (pH = 8.5) at room temperature and washed with PBS 3x. Sections were blocked in 10% donkey serum in 0.25% PBS + Trition for 1 h at room temperature. Sections were then incubated overnight at 4^0^ C with primary antibody FHL2 (anti-rabbit, Genetex, 1:100) and DCX (anti-goat, Santa Cruz Biotechnology 1: 500), BrdU (anti-rat, Abcam 1: 1000), Hes5 (anti-goat, Santa Cruz Biotechnology 1: 500), Ki67 (anti-ms, Abcam 1: 250) in 0.25% PBST plus 10% donkey serum. Following day, sections were washed 3x with 0.25% PBST and were then incubated in secondary antibodies (Alexa 488 anti-rabbit, 1:100, 594 anti-mouse and 674 anti-goat, 1:500) at room temperature for 3 h. Next, sections were washed 3x with PBS and were mounted on a slide and coverslip with DAPI for confocal analysis.

### Imaging and Quantification

All coronal sections were imaged on an Olympus FLV1000 confocal microscope. The Z stack confocal images of the different regions of the brain including DG, CA3, CA2, and CA1 regions were collected and the fluorescence intensity was measured using Image J software. The fluorescence intensity of FHL2 of the whole cell was measured by taking the difference between the fluorescent intensity of the cell and background signal from the same image. The morphometric profile used for the neuron selection for analysis includes bipolar feature of the anatomical phase of the cell, the elliptical cell body and approximately 10 μm size of the cell (Stanfield and Cowan, 1979; Claiborne et al., 1990; Danzer et al., 2008) and immunolabeling with Prox1 marker. Dendritic tracings were constructed using Imaris software (Oxford Instruments). Sholl analysis was performed using ImageJ software, dendritic branches were measured from soma at 5 μm interval.

### Statistical Analyses

Data were analyzed with independent samples *t-*tests and one-way ANOVA followed by *post hoc* Tukey HSD test. Two-tailed values of α < 0.05 were considered the cutoff for statistical significance. All data are represented as mean ± SEM. N represents the number of animals unless otherwise specified.

## Results

### Endogenous Expression of FHL2 in the Adult Hippocampus

**FIGURE 1:**
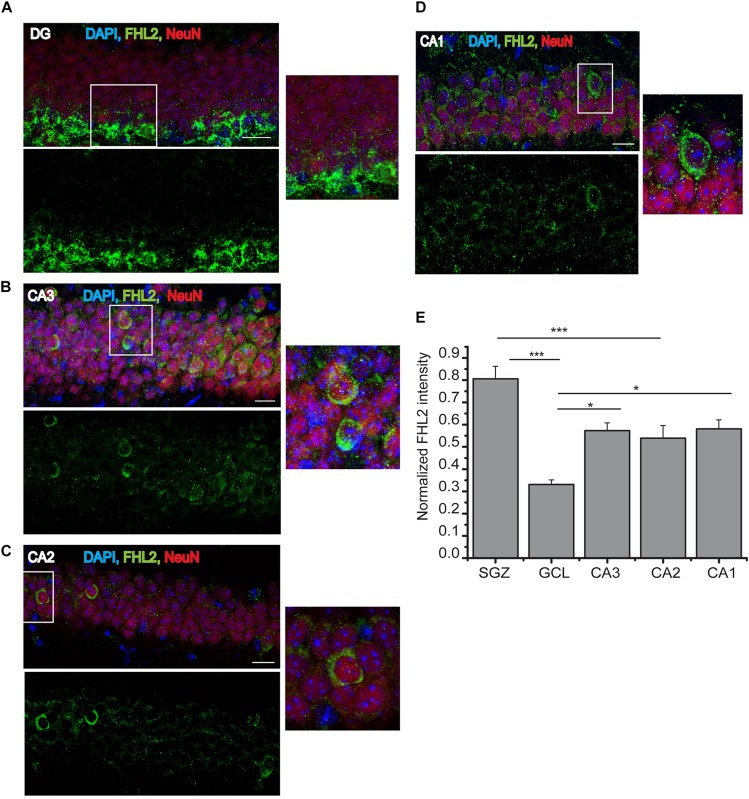
Endogenous expression of FHL2 in the adult hippocampus. **(A–D)** Representative images of hippocampal subfields dentate gyrus (DG), CA3, CA2, and CA1 (respectively), immunolabeled for FHL2 and NeuN. **(E)** Quantification of normalized fluorescence of FHL2 throughout the hippocampus. One-way ANOVA *F*(3,156) = 5.69, *P* = 0.001. Scale bars, 10 μm, *N* = 3 mice per condition. Data are presented as mean ± SEM. ***p < 0.001 and ****p < 0.0001.

In order to study the regulatory role of FHL2 in the adult brain, we first mapped the expression of FHL2 in the subfields of the adult hippocampus [dentate gyrus (DG), CA3, CA2, and CA1] (Figures 1A–D). We observed whole-cell fluorescence intensity of FHL2 throughout the neurons of these regions, with no significant differences between regions CA3, CA2 and CA1 (*p* > 0.05), however, significant differential expression was observed between the sub-granular zone (SGZ) and granular cell layer (GCL) (*p* < 0.001), CA3 and GCL (*p* = 0.019), CA2 and SGZ (*p* = 0.001), CA1 and GCL (*p* = 0.016) (Figure 1E). Thus, FHL2 is expressed throughout the adult hippocampus, consistent with previous observations in the embryonic central nervous system (CNS) (Kudo et al., 2007).

### Expression of FHL2 in Neural Stem Cells, Proliferative Cells and Mature Granule Cells

**FIGURE 2:**
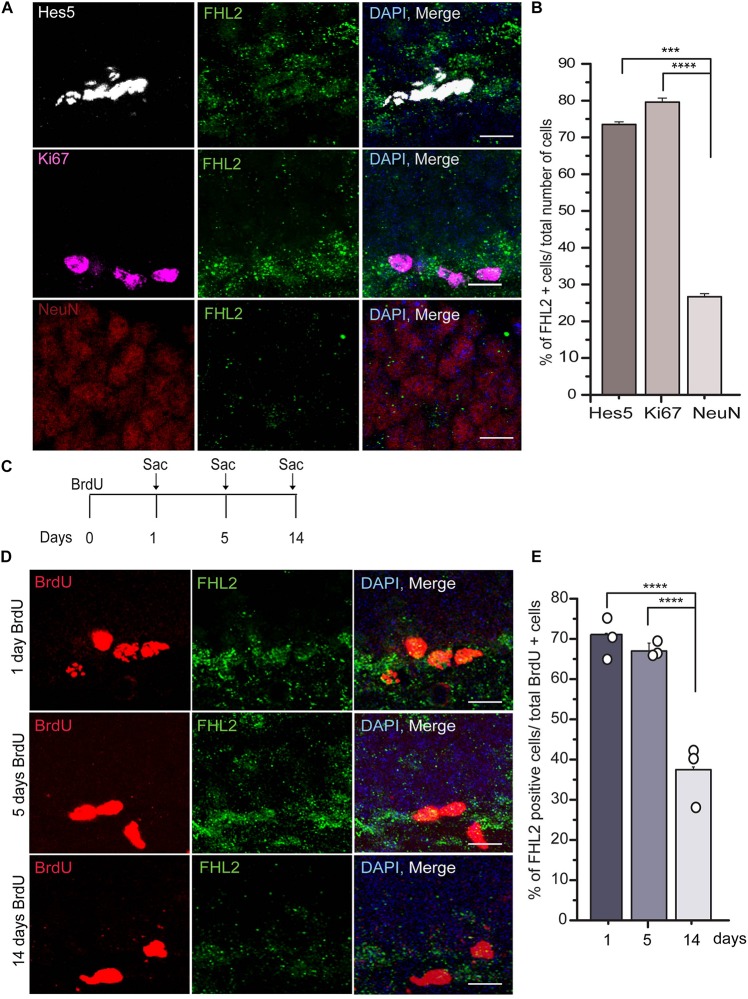
FHL2 expression across developmental time in the dentate gyrus. **(A)** Representative images of Hes5, Ki67, and NeuN positive with FHL2 colocalization. **(B)** Quantification of the percentage of FHL2 positive cells in a total number of Hes5, Ki67 and NeuN positive cells. One way ANOVA *F*(2,87) = 10.399, *P* < 0.001. Followed by *post hoc* Tukey HSD test, Hes5 vs. Ki67 (*p* > 0.05), Hes5 vs. NeuN (*p* = 0.001) and Ki67 and NeuN (*p* < 0.001). **(C)** Timeline for BrdU injection to label the proliferating cells. **(D)** Representative images of BrdU positive cells colocalized with FHL2 at 1 day, 5 days, and 14 days post-injection. **(E)** Quantification of colocalization of FHL2 with BrdU positive cells at 1, 5, and 14 days after injection. One way ANOVA *F*(2,31) = 14.861, *P* > 0.001. Followed by *post hoc* Tukey HSD test, BrdU d1 vs. BrdU d5 (*p* > 0.05), BrdU d1 vs. BrdU 14 (*p* < 0.001) and BrdU d5 vs. BrdU14 (*p* < 0.001). Data are presented as mean ± SEM. Scale bar, 10 μm. *N* = 3 mice per condition.

Next, we sought to determine whether the expression of FHL2 changes as a function of developmental time for maturing cells in the DG. We measured the number of FHL2 positive cells in the population of cells expressing *Hes5*, a marker of neural stem cells (NSCs), Ki67, a proliferative marker, and NeuN, a marker of differentiated neurons in the granule cell layer of the DG (Figure 2A). The majority of Hes5 + cells (73.5 ± 0.75%) and Ki67 + cells (79.6 ± 1.13%) positive cells express FHL2 under basal conditions, while the expression of FHL2 is significantly lower in NeuN + neurons (26.7 ± 0.79%) (Figure 2B). Next, we birthdated the dividing cells by injecting the thymidine analog BrdU intraperitoneally and euthanized the animals at 1, 5, and 14 days after injection and quantified the number of cells positive for FHL2 expression over three-time points (Figures 2C,D). We found that the percentage of FHL2 positive cells declined over time, day 1 (71.1 ± 2.6%), 5 days (67.0 ± 1.97%), and 14 days (37.5 ± 0.65%) (Figure 2E). Thus, our data suggest based on the expression pattern of FHL2 in the majority of stem cells and proliferative cells and decreased expression in neurons as they mature that FHL2 may play a regulatory role in modulating the early phase of the cell programming.

### Expression and Genetic Manipulation of FHL2 in Adult-Born DGCs

**FIGURE 3:**
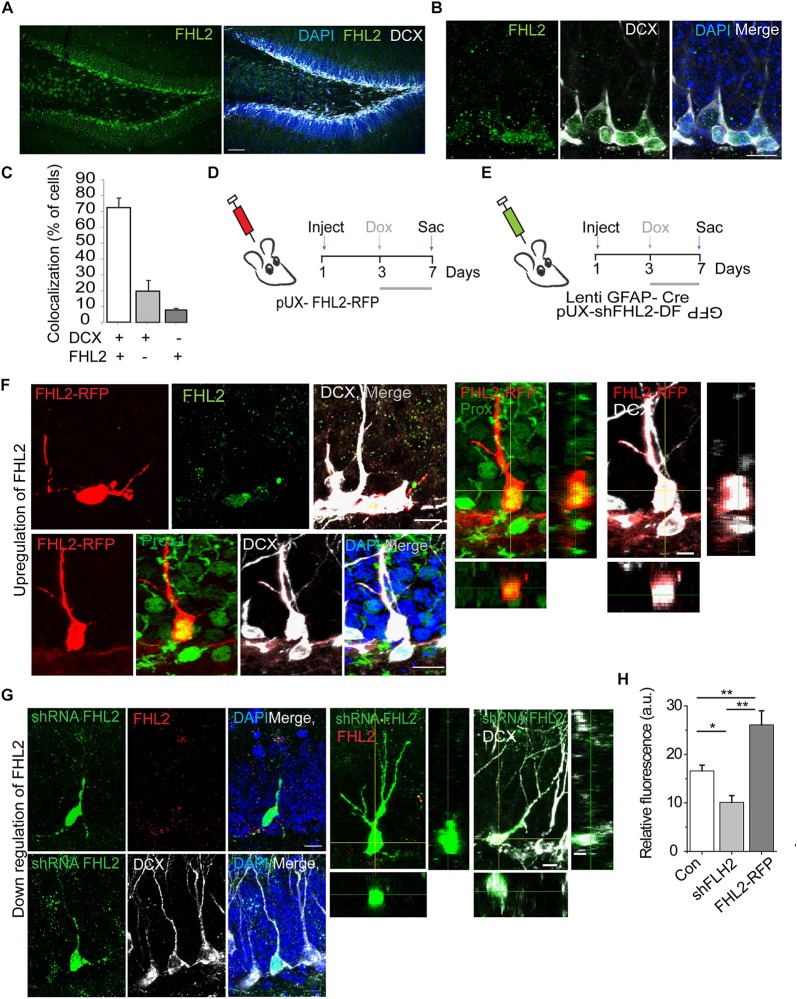
Endogenous expression and successful manipulation of FHL2 in adult-born DGCs. **(A)** Representative coronal section of the DG immunolabeled with FHL2 and DCX. Scale bar, 100 μm. **(B)** Representative image of the dentate gyrus labeled with FHL2 colocalized with DCX. Scale bar, 10 μm. **(C)** Quantification of colocalization between DCX and FHL2 in the dentate gyrus. **(D)** Experimental timeline of inducible overexpression of FHL2 in adult-born DGCs with doxycycline induction (dox) at 3 dpi and sacrifice (sac) at 7 dpi. **(E)** Experimental timeline of inducible downregulation of FHL2 in adult-born DGCs with doxycycline induction (dox) at 3 dpi and sacrifice (sac) at 7 dpi. **(F)** Representative image of a newborn neuron at 7 dpi overexpressing FHL2 with FHL2 signal shown (top) and Prox 1 colocalization shown (bottom). Orthogonal views of the expression of Prox1 and DCX in RFP positive cells shown in the right panels. **(G)** Representative images of newborn neurons (DCX +) at 7 dpi with viral-mediated FHL2 knock-down. Orthogonal analysis of the expression of FHL2 and DCX colocalization shown in the right panels. **(H)** Quantification of relative fluorescence of FHL2 signal in control (con) newborn DGCs, shFHL2 cells, and FHL2-RFP cells (*n* = 103 cells) at 7 dpi. One-way ANOVA *F*(2,67) = 15.64, *P* < 0.0001. Followed by *post hoc* Tukey HSD tests, con vs. shFHL2, *P* = 0.037; con vs. FHL2-RFP, *P* < 0.01; shFHL2 vs. FHL2-RFP, *P* < 0.01, *P < 0.05, and **P < 0.01. Error bars, SEM. *N* = 3–4 mice per condition. Data are presented as mean ± SEM, Con, control.

Having established that FHL2 is expressed in the adult hippocampus including the DG, we next sought to determine whether FHL2 is expressed in newborn hippocampal neurons. For this purpose, we stained adult brain sections for FHL2 and doublecortin (DCX), a marker of immature neurons in the migratory phase. The majority (72.5 ± 5.9%) of DCX positive cells co-localized with FHL2, while a small fraction (19.7 ± 6.7%) of FHL2 + cells were DCX negative and a smaller subset (7.8 ± 1.8%) of DCX positive cells were negative for FHL2 (Figures 3A-C). To evaluate the physiological role of FHL2 in adult-born DGCs, we constructed inducible overexpression and knockdown of FHL2 vectors packaged into retroviruses (Figures 3D,E). In order to avoid potential effects on cell fate determination (Kim et al., 2019), we induced expression with doxycycline from day 3, as shown in Figures 3D,E. We observed that all FHL2-RFP positive cells expressed Prox1 and DCX (Figure 3F) thus validating our experimental approach to target adult-born neurons without affecting cell fate choice. We also confirmed that FHL2-knockdown cells (83.9 ± 0.9%) exhibited reduced FHL2 fluorescence signal intensity (Figure 3G) as compared to DCX positive internal control cells, while FHL2-overexpressing cells (78.7 ± 1.61%) exhibited heightened FHL2 fluorescence signal intensity as compared to such control cells (Figure 3H). Together, these data demonstrate the establishment of a temporally specific, bidirectional genetic manipulation approach to study the function of FHL2 in adult-born DGCs under basal conditions.

### Overexpression of FHL2 Expands Dendritic Outgrowth in Adult-Born Dentate Granule Cells

**FIGURE 4:**
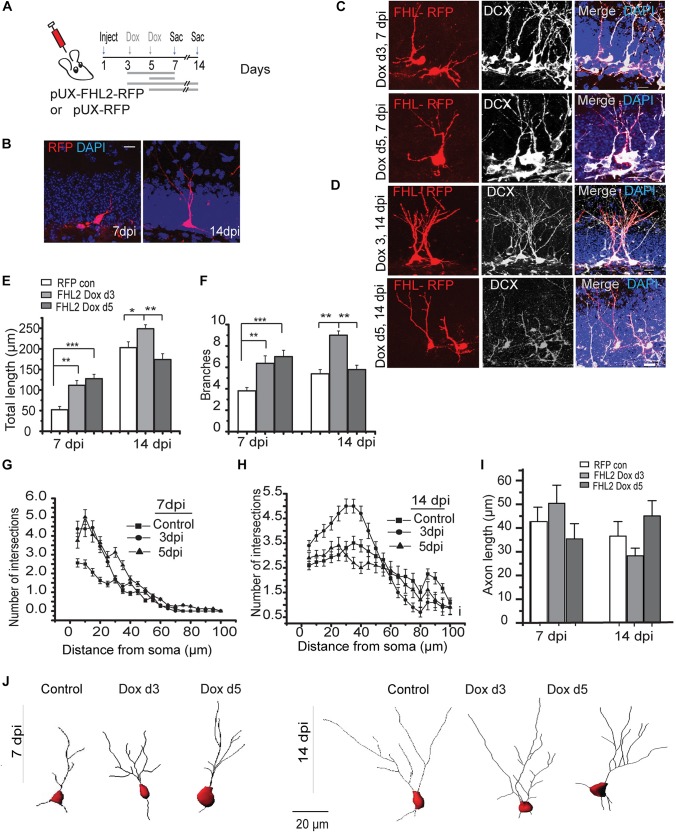
Overexpression of FHL2 expands dendritic outgrowth in adult-born DGCs. **(A)** Experimental timeline of inducible FHL2 overexpression, with doxycycline induction (dox) at 3 or 5 dpi and sacrifice (sac) at 7 or 14 dpi. **(B)** Representative images of control DGCs expressing RFP (RFP con) at 7 and 14 dpi. Scale bar, 10 μm. **(C)** Representative images of FHL2-overexpressing cells co-labeled with DCX at 7 dpi, induced at either 3 dpi (top) or 5 dpi (bottom). Scale bar, 10 μm. **(D)** Representative images of FHL2-overexpressing cells co-labeled with DCX at 14 dpi, induced at either 3 dpi (top) or 5 dpi (bottom). Scale bar, 10 μm. **(E)** Quantification of total dendrite length at 7 and 14 dpi for control cells expressing RFP only, and FHL2-overexpressing cells induced at 3 or 5 dpi (*n* = 116 cells). 7 dpi: One-way ANOVA *F* (2,45) = 9.162, *P* < 0.0001, followed by *post hoc* Tukey HSD test: RFP con vs. FHL2 Dox d3, *P* < 0.01; RFP con vs. FHL2 Dox d5, *P* < 0.001. 14 dpi: One-way ANOVA *F* (2,120) = 8.58, *P* < 0.0001, followed by *post hoc* Tukey HSD test: RFP con vs. FHL2 Dox d3, *P* < 0.05; FHL2 Dox d3 vs. FHL2 Dox d5, *P* < 0.01. **(F)** Quantification of number of dendritic branch points at 7 and 14 dpi for control cells expressing RFP only, and FHL2-overexpressing cells induced at 3 or 5 dpi (*n* = 116 cells). 7 dpi: One-way ANOVA *F* (2,45) = 9.162, *P* < 0.0001, followed by *post hoc* Tukey HSD test: RFP con vs. FHL2 Dox d3, *P* < 0.01; RFP con vs. FHL2 Dox d5, *P* < 0.001. 14 dpi: One-way ANOVA *F* (2,120) = 26.79, *P* < 0.0001, followed by *post hoc* Tukey HSD test: RFP con vs. FHL2 Dox d3, *P* < 0.01; FHL2 Dox d3 vs. FHL2 Dox d5, *P* < 0.01. **(G)** Sholl analysis at 7 dpi. Two-way ANOVA *F* (2, 44) = 6.814, *p* = 0.003, con vs. FHL2 Dox d3, *p* = 0.05, control vs. FHL2 Dox d5, *p* = 0.02, FHL2 Dox d3 vs. FHL2 Dox d5 *p* = 0.427. **(H)** Sholl analysis at 14 dpi: Two-way ANOVA *F* (2, 120) = 4.107, *p* = 0.01, followed by *post hoc* Tukey HSD test: RFP con vs. FHL2 Dox d3, *p* = 0.036, control vs. FHL2 Dox d5, *p* > 0.05, FHL2 Dox d3 vs. FHL2 Dox d5 *p* < 0.05. **(I)** Quantification of axonal length at 7 and 14 dpi for control cells expressing RFP only, and FHL2-overexpressing cells induced at 3 or 5 dpi. 7 dpi: One-way ANOVA *F* (2,57) = 1.23, *p* = 0.299.14 dpi: One-way ANOVA *F(*2,57) = 2.38, *p* = 0.102. *N* = 3–4 mice per condition. Data are presented as mean + SEM. **(J)** Representative dendritic tracing of control cells expressing RFP only and FHL2-overexpressing cells dox d3 and dox d5 at 7- and 14 dpi, scale bar, 20 μm.

Dendritic elaboration in adult-born DGCs begins at approximately 1 week after birth, with this phase in DGC maturation accelerating by 2 weeks (Zhao et al., 2006; Goncalves et al., 2016; Wang et al., 2019), at a developmental time when we observed endogenous FHL2 expression (Figure 3). This led us to hypothesize that FHL2 may play a role in dendrite outgrowth during a critical period in the initial development of nascent DGCs. To determine whether FHL2 affects the dendritic growth of developing DGCs, we induced overexpression of FHL2 in the dividing hippocampal cells at either 3 or 5dpi as illustrated in Figure 4A and sacrificed mice at 7 and 14 dpi. As a control, a retroviral vector carrying only an RFP tag was injected, and mice were treated with doxycycline from day 3 and sacrificed at 7 or 14 dpi (Figures 4A,B). Among cells overexpressing FHL2 from 3 dpi, such overexpression expanded dendrite length and branching at 7 dpi, an effect that persisted at 14 dpi (Figures 4C–F). Interestingly, in cells that had been induced at 5dpi, there was a similar increase in total dendrite length and branching at 7dpi, however, this effect was short-lived, reverting to the wildtype phenotype by 14dpi (Figures 4C–F). Sholl analysis revealed increased dendritic complexity in the experimental groups at 7 and 14 dpi as compared to the control group (Figures 4G,H). In addition, we wondered whether FHL2 overexpression would affect axonal length at 7 and 14 dpi, but found no significant differences at any time point compared to control (Figure 4I). Representative cell traces in the various conditions are shown in Figure 4J. Together, these data demonstrate that FHL2 overexpression expands dendritic outgrowth in newborn DGCs at an early developmental stage.

### Loss of FHL2 Increases Early DGC Dendritic Length

**FIGURE 5:**
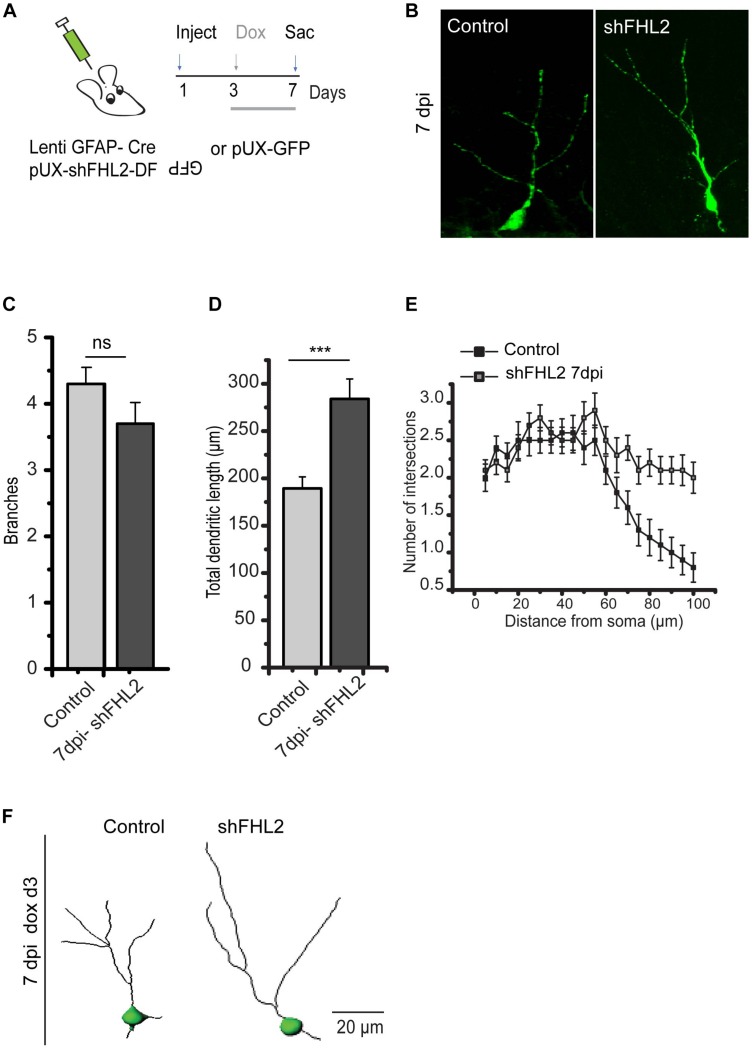
Silencing of FHL2 increases adult-born DGC dendritic length during early development. **(A)** Experimental timeline of inducible FHL2 down-regulation with doxycycline induction (dox) at 3 dpi and sacrifice (sac) at 7 dpi. **(B)** Representative images of GFP control (GFP con) and shFHL2-expressing DGCs at 7 dpi induced at 3 dpi. Scale bar, 10 μm. **(C)** Quantification of the number of dendritic branches in GFP con and shFHL2-expressing DGCs at 7 dpi. Two-tailed unpaired *t*-test, *p* > 0.05. **(D)** Quantification of total dendrite length for GFP-only expressing control and shFHL2 cells at 7 dpi. Two-tailed unpaired *t*-test, at 7 dpi, *p* < 0.05. **(E)** Sholl analysis at 7 dpi. Two-way ANOVA *F* (1, 56) = 925.96, *p* < 0.001. **(F)** Representative dendritic tracing of control cells and shFHL2 expressing cells dox induced d3 at 7 dpi, scale bar, 20 μm.

Given that FHL2 expression, especially during an early critical window immediately after fate determination, is important for dendritic arborization, we next sought to understand the implications of its loss on the maturation in DGCs, which may ultimately influence their functional integration (Kuipers et al., 2009; Ma et al., 2009; Krueppel et al., 2011; Bergami et al., 2015). To this end, we first co-injected lenti-GFAP-Cre and retro-shFHL2-DF-rGFP in the hippocampus to target active radial glia-like (RGL) cells for subsequent FHL2 downregulation (Figure 5A). We induced expression at 3 dpi, as we had found this early time point to be critical for dendritic elaboration (Figure 4). Loss of FHL2 in newborn DGCs did not affect dendritic branching, but significantly increased dendritic length (Figures 5B–F). These results suggest that loss of FHL2 in newborn neurons has a distinct yet overlapping effect as gain. Of note, we examined the effect of FHL2 downregulation at 14 dpi as well, but were unable to establish a clear, reproducible phenotype (data not shown). Taken as a whole, early expression of FHL2 in adult-born DGCs appears to regulate appropriate early morphological growth, ensuring that dendrites do not “overshoot”, with implications for these cells’ proper integration.

## Discussion

Dendritic development, a process essential for DGC maturation and plasticity of the hippocampal network, is tightly regulated by molecular machinery during the course of dentate granule cell growth. Several mechanisms regulate the initiation, formation, and maintenance of dendritic arborization such as microtubule nucleation and microtubule-associated motor protein (Sharp et al., 1997; Delandre et al., 2016), secretory pathways involving the Golgi apparatus (Rao et al., 2018) and endoplasmic reticulum (Cui-Wang et al., 2012), and synaptic scaffolding proteins (Vessey and Karra, 2007) suggesting that the sculpting and maintenance of the dendritic tree has many layers of complexity. Although the dysregulation of dendritic development is reported in MTLE (Curia et al., 2014) the precise mechanism underlying the pathophysiology of MTLE remains uncertain. Interestingly *FHL2*, a gene linked to cellular metabolism has been found to be dysregulated in MTLE, although the reason for this is unknown (Tran et al., 2016). In this study, we found that manipulation of FHL2 disrupts dendritic modeling in newborn DGCs in the hippocampus by causing dendritic hypertrophy, a phenotypic recapitulation of neurons of patients with MTLE which points to a potential underlying mechanism (Ryufuku et al., 2011).

FHL2, a LIM domain protein has a unique affinity for various intracellular proteins and receptors such as in the context of regulation of bone marrow-derived dendritic cell migration through its Sphingosine 1-phosphate receptor (S1PR1) interaction (König et al., 2010). FHL2 also interacts with a microtubule-associated protein 1 light chain 3 (LC3) regulating the development of skeletal muscle cells (Liu et al., 2019), and with β-catenin to upregulate its transactivation activity in cancer cells (Wei et al., 2003). Thus, FHL2 participates in various protein complexes regulating cell growth, differentiation and cytoskeletal remodeling as reported in immune cells and in the pathogenesis of numerous cancers (Canault et al., 2006; Wixler et al., 2007; Li et al., 2008; Qian et al., 2009; Kurakula et al., 2014). Although several studies have shed light on the relevance of FHL2 in terms of CNS-related pathology, including epilepsy and glioblastoma (Jamali et al., 2006; Kleiber et al., 2007; Li et al., 2008; Okamoto et al., 2013), the understanding of the regulatory role of FHL2 is yet to be fully appreciated. Recently, Kim et al. (2019) reported that FHL2 is expressed by NSCs and plays a role in both fate determination of cells as well as the migratory capabilities of their progeny cells (Kim et al., 2019). To assess the physiological implication of FHL2 on the dynamic process of dendritic arborization, we report for the first time that FHL2 is expressed endogenously in adult-born hippocampal neurons and interestingly that there is a significant decline in its expression observed as these neurons mature, suggesting the participation of FHL2 during the early development of adult-born neurons. We used viral vector-mediated manipulation of FHL2 expression at the selected time point to avoid any confounding effect on fate determination (Kumamoto et al., 2012) and successfully targeted the cells committed to the neuronal lineage (Figure 3). Here we found the paradoxical effects of upregulation and downregulation of FHL2, which both increased the dendritic growth during the early developmental phase of DGCs. One plausible reason could be that levels of FHL2 required for proper dendritic outgrowth follow a “U shaped curve”, with higher or lower levels both leading to loss of inhibition of dendritic expansion, although this remains to be tested. We also did not observe a consistent phenotype of downregulation of FHL2 on dendritic morphogenesis at the later developmental time point of 14 dpi (data not shown), which may relate to effects of low levels of FHL2 on cell viability at the early survival/synaptic integration stage. Nevertheless, our findings suggest a contribution of early expression of FHL2 in DGC morphogenesis, which may be explained by various signaling partner interactions.

One candidate pathway by which FHL2 may participate in DGC dendritic morphogenesis is the sphingolipid signaling pathway. Based on FHL2 interactome data (Tran et al., 2016), Sphingosine Kinase (SphK) is one of the metabolism-linked interacting partners of FHL2 and regulates the availability of Sphingosine 1 Phosphate (S1P), a bioactive lipid metabolite, reported to be involved in cell proliferation, survival, migration and cytoskeletal remodeling (Spiegel and Milstien, 2003). S1P exerts its downstream effects by binding to one of the substrate receptors, S1PR1, highly expressed on new adult-born DGCs to modulate neurite growth (Toman et al., 2004; Hayashi et al., 2009; Yang et al., 2020). Yet, whether FHL2 participates in sphingolipid signaling in this context will require further molecular characterization. Alternatively, the influence of FHL2 on dendritic modeling could be explained through interactions with the Wnt/β-catenin signaling pathway (Martin et al., 2002), which has been shown to refine dendrites in newborn DGCs (Kumamoto et al., 2012) and is also dysregulated in some forms of epilepsy (Hodges and Lugo, 2018).

In this study, we focused on the acute effects of FHL2 expression on early DGC development, however, there may be differential effects of chronic upregulation and blockade of FHL2 expression on dendritic growth in the adult brain, which will require further exploration. Based on the diverse interaction profile of FHL2 and its affinity to form protein-protein complexes as part of a signal interaction node, there remains much to be gleaned regarding the mechanisms governing dendritic modeling as well as dendritic hypertrophy and synaptic perturbation reported in MTLE (Ryufuku et al., 2011; Han et al., 2016; Janz et al., 2018). While we limited the scope of the present study to focus on dendritic alteration, FHL2 may play roles in other aspects of DGC morphogenesis and physiology. In summary, we have shown that FHL2 is an important regulator of early dendritic morphogenesis in adult-born hippocampal neurons. This study serves as a new line of evidence demonstrating the biological relevance of FHL2 in the central nervous system.

## Data Availability Statement

The datasets generated for this study are available on request to the corresponding author.

## Ethics Statement

The animal study was reviewed and approved by Stony Brook University Animal Use Committee.

## Author Contributions

JW and SG conceived of the idea. JW engineered the vectors. AA, JW, and GK conducted the experiments. AA and GK wrote the initial draft. All authors analyzed the data and agreed with the final version of the manuscript.

## Conflict of Interest

The authors declare that the research was conducted in the absence of any commercial or financial relationships that could be construed as a potential conflict of interest.
